# The Airman’s Edge Project: A Peer-Based, Injury Prevention Approach to Preventing Military Suicide

**DOI:** 10.3390/ijerph18063153

**Published:** 2021-03-18

**Authors:** Justin C. Baker, Craig J. Bryan, AnnaBelle O. Bryan, Christopher J. Button

**Affiliations:** 1 Department of Psychiatry and Behavioral Health, The Ohio State University Wexner Medical Center, Columbus, OH 43210, USA; craig.bryan@osumc.edu (C.J.B.); annabelle.bryan@osumc.edu (A.O.B.); 2 509th Operational Medical Readiness Squadron, Knob Noster, MO 65305, USA; christopher.j.button.mil@mail.mil

**Keywords:** peer mentoring, suicide, military, peer-to-peer, occupational safety

## Abstract

In light of data indicating military personnel are more likely to reach out to peers during times of need, peer-to-peer (P2P) support programs have been implemented for military suicide prevention. Often designed to reduce suicidal thoughts and behaviors by reducing mental health symptom severity, existing data suggest that P2P programs have little to no effect on mental health symptoms. Conceptualizing suicide prevention from an occupational safety and injury prevention perspective to promote positive health-related behavior change at both the group and individual level may enhance the effectiveness of P2P programs and military suicide prevention efforts more broadly. To illustrate these concepts, the present article provides an overview of the Airman’s Edge project, a P2P program design based upon the occupational safety and injury prevention model of suicide prevention, and describes a program evaluation effort designed to test the effectiveness of this approach.

## 1. Introduction

Suicide among U.S. military members has steadily increased since 2004 across all branches of service [1,2]. In the U.S. Air Force, the suicide rate among active duty Airmen nearly doubled from 2001 to 2015, increasing from 9.1/100,000 to 20.5/100,000 in 2015 [1,2]. Thinking about killing oneself is also higher with 18 percent of military members reporting having these thoughts at some point in their lives compared to only 4 percent of the general population reporting similarly, according to the 2015 Department of Defense Health Related Behaviors Survey [3]. In response, the Department of Defense (DoD) has invested heavily in identifying, developing, and testing interventions to reverse this trend, resulting in several recent advances including brief cognitive behavioral therapy (BCBT) for suicide prevention [4] and crisis response planning [5], each of which contribute to significant reductions in suicidal behaviors among military personnel as compared to treatment as usual. Though promising, these evidence-based interventions are only available to service members from specially trained mental health professionals working within military treatment facilities. Fewer than 25% of military suicide decedents receive outpatient mental health services or substance abuse services within the 90 days preceding their deaths [2], however, suggesting the reach and overall impact of these treatments on military suicide would be modest at best. Strategic suicide prevention interventions that target health-related behaviors specific to a military population and extend beyond mental healthcare settings, are therefore needed. 

Military personnel experiencing mental health issues are more likely to reach out to peers than healthcare professionals [6], leading some to propose peer-to-peer (P2P) programs for the purposes of community-based suicide prevention [7]. Peer-based interventions have often been used to promote important health-related behavior change [8]; however, empirical support for this approach is lacking. One P2P program implemented in a non-military medical setting found that patients who received peer-based counseling had significantly lower rates of suicidal behaviors during follow-up as compared to patients who received treatment as usual [9]. Though promising, that program’s placement within a medical setting and emphasis on offering therapeutic interventions limits generalizability to community-based P2P models that emphasize peer support, wherein peer mentors offer informal support in the form of reminders about appointments, encouragement, and sharing personal experiences as a “buddy,” but do not develop formal therapeutic relationships [10].

Although the effects of P2P programs on suicidal thoughts and behaviors have not been examined, a recent review of 116 randomized controlled trials examining the effects of P2P programs on a range of other outcomes including attitudes and beliefs (including stigma, help-seeking intentions), behavior change, social connectedness, and mental health have been considered [10]. The results of this review suggest that peer educator models, wherein peers provide formal education or training on specified topics using protocolized curricula without a therapeutic relationship, tended to positively change attitudes and beliefs and enhance social connectedness. This effect was more likely to be observed when the peer education was delivered as a group intervention than a dyadic (i.e., peer-to-peer) intervention. In contrast, dyadic interventions were more likely than group interventions to positively change specific health-related behaviors when P2P programs adopted peer support models wherein peers provided informal and unstructured support to others. Peer educator models also had a positive impact on target behaviors when delivered in a hybrid model that combined elements of the group and dyadic formats.

In the above mentioned review of 116 randomized controlled trials examining P2P programs, no particular design feature was consistently associated with improved mental health outcomes [10]. Although some studies supported positive mental health outcomes associated with P2P programs that used group peer educator or dyadic peer support interventions, 2–3 times as many studies reported no benefit. Consistent with these patterns, a military-based P2P program utilizing a peer support model recently reported no change in depression or PTSD symptom severity during the years that coincided with program implementation [7,11]. In combination these patterns suggest that P2P program effectiveness depends in part on how it is designed, how it is implemented, and the outcomes targeted. As a strategy for suicide prevention, P2P programs may be most effective when designed and implemented in a manner that maximizes the probability of changing behaviors rather than changing mental health symptoms and/or attitudes and beliefs.

In this paper, we will describe an alternative approach to P2P program development and implementation for the purposes of suicide prevention that is based on an occupational safety and workplace injury prevention model. We first discuss how an occupational safety and injury prevention approach might be applied to the military community, then describe the Airman’s Edge project, a P2P program designed for the purposes of reducing suicidal behaviors among military personnel based on an injury prevention rather than a mental health model. Finally, we provide details about an ongoing effort to evaluate the program’s effects on suicidal thoughts and behaviors, and highlight several design considerations to address common challenges associated with evaluating the effects of community-level programs on promoting positive health-related behavior change within an organization.

## 2. An Occupational Safety Model of Suicide Prevention

**Figure 1:**
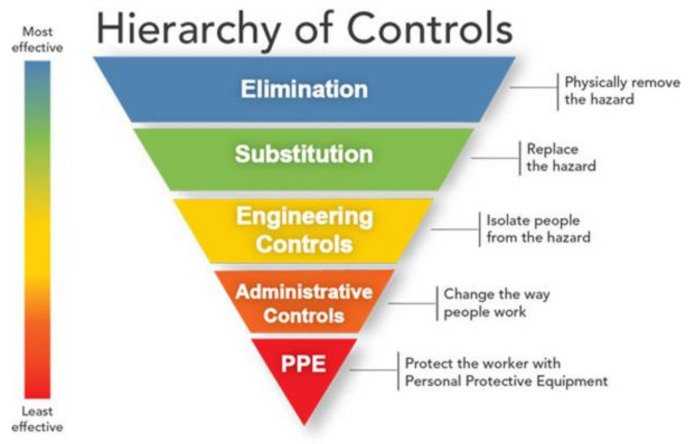
Hierarchy of Controls. Based off the National Institute for Occupational Safety and Health national initiative to reduce or completely eliminate workplace injuries, illnesses, and fatalities [12]. The presented hierarchy argues that intervening at the top of the model is likely more effective than those presented at the bottom.

Central to workplace injury prevention programs is the “hierarchy of controls” concept (see Figure 1), which serves to rank order the potential effectiveness of strategies intended to reduce the risk of illness or injury [12]. At the top of this hierarchy, coinciding with the highest level of effectiveness, are strategies that seek to physically remove a hazard from the environment. At the bottom of the hierarchy, coinciding with the lowest level of effectiveness, are strategies that seek to protect individual workers from a hazard. Removal of a hazard is the most effective strategy because it eliminates exposure to the very thing that increases risk to the worker, and is not reliant on individual behavior change. Elimination strategies also maximize effectiveness because they can potentially remove or reduce risk for many (potentially all) workers. By comparison, strategies that seek to protect workers from a hazard that remains in the environment are less effective because the risk for illness or injury persists, and protection from this risk depends upon the sustained integrity of the protective strategy as well as worker adherence (e.g., using the protective strategy correctly and consistently).

Unfortunately, complete elimination of an environmental hazard is not always possible. According to the hierarchy of controls model, the next most effective solution entails substitution, wherein a hazard is replaced with a less dangerous hazard, thereby incrementally reducing the risk of illness or injury. For example, polyurethane foams and cellulose fibers are common building material alternatives to asbestos, thereby reducing health risks associated with asbestos exposure. When elimination or substitution is not feasible, however, engineering controls may be employed next, thereby isolating or otherwise separating workers from a hazard. Sections of a building or a community may, for instance, be sealed off during a mishap to restrict people’s access to a potential hazard. Next, administrative controls can be used to minimize the extent to which workers are exposed to the hazard. Administrative controls include safety-focused practices and procedures like pre-flight checklists, 101 Critical Days of Summer safety briefs, and rifle range safety rules. Finally, personal protective equipment (PPE) can be used to minimize the likelihood of illness or injury despite ongoing exposure to the hazard, such as distributing and using gas masks and mission oriented protective poster (MOPP) gear, or mandatory helmet use when riding a motorcycle. An occupational safety and injury prevention approach therefore mirrors the “culture of safety” principles and practices that are already embedded within many areas of daily military life.

As applied to suicide prevention, mental health treatments are best understood as a form of PPE because they primarily serve to protect the individual from hazards that are not well controlled. For example, service members who receive BCBT for suicide prevention must continue to implement the skills learned in therapy for an indefinite length of time, potentially well after treatment ends [4]. BCBT and other suicide-focused treatments are not designed to alter or change environmental conditions, however, meaning that service members who receive the treatment may be continuously exposed to circumstances that fuel their suicidal desire. The protective effects of BCBT therefore depend in part on the durability of the treatment, which depends in part on the reliability with which the treatment was delivered by the clinician (i.e., treatment fidelity) and in part on the extent to which the service member uses these concepts in their lives. By comparison, eliminating or reducing the service member’s exposure to the conditions that sustain their suicide risk could reduce the probability of suicidal behavior regardless of treatment engagement.

The majority of the resilience training programs that have been implemented within the military are also reasonably categorized as PPE because they aim to protect the service member from psychological hazards without removing or altering the source of these hazards. Even though resilience programs are often implemented at the group level and align with P2P program models (e.g., educational classes may be taught by specially trained peers or “master resilience trainers”), the central target of action for these programs nonetheless lie within individual service member rather than the environment within which the service member works and lives. As a result, the potential effectiveness of these “upstream” approaches are much more limited than often assumed.

The effectiveness of P2P programs could potentially be enhanced by shifting their focus to higher levels of the control hierarchy and adopting a “prevention through design” approach [12]. Although the prevention through design mind-set is most often associated with workplace safety and injury prevention methods, the key concepts of this approach can be readily extended to non-workplace settings as well (e.g., residences, personally owned vehicles). The Airman’s Edge P2P program was designed based on these principles, and is described here.

## 3. The Airman’s Edge Peer-to-Peer (P2P) Program

**Figure 2:**
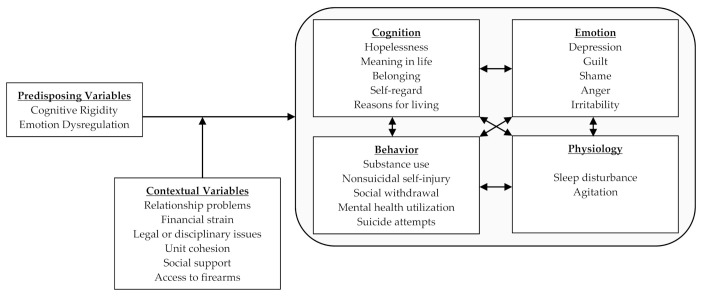
The Suicidal Mode.

The Airman’s Edge project is a Department of Defense-funded suicide prevention effort aimed at evaluating the effectiveness of a P2P program that was designed to reduce suicidal behaviors. In contrast to typical approaches to suicide prevention that conceptualize suicide as an individual-level problem closely associated with mental illness and psychological well-being, Airman’s Edge conceptualizes suicide from an injury prevention perspective. Specifically, we conceptualize suicide risk as outlined below in The Suicide Mode (see Figure 2). The suicidal mode entails two facets: internal factors (cognition, behavior, emotion, and physiology) and external/contextual factors (life stressors, unit cohesion, access to firearms, etc.). According to this model, the suicide mode outlines the probability that an individual will attempt suicide in response to contextual and/or environmental factors is therefore conditioned upon their predisposing vulnerabilities, namely cognitive rigidity and emotion dysregulation. Traditional mental health treatment focuses on changing and altering these internal factors. In the proposed injury prevention model, the focus is to change the external factors such that people with many internal factors are less likely to experience activation of the suicidal mode. Consistent with this approach, Airman’s Edge seeks to leverage peers to encourage and promote positive health-related behavior change directly within the environments military personnel live using a combination of elimination, engineering, and administrative controls.

### 3.1. Selection of Peer Mentors

Peer mentors are selected via a process informed by Defense Centers of Excellence best practices recommendations [6], wherein squadron leaders and service members nominate individuals whom they perceive as possessing the following skills and attributes: communication and listening skills, demonstrated leadership ability or potential, ability to remain calm under pressure, ability to effectively conduct briefings and/or public presentation, and previous experience and training. Peer mentors are selected with consideration for gender, rank, and racial/ethnic background in order to increase the diversity of peer mentors and the likelihood that other service members will feel comfortable approaching a peer mentor. Due to mission demands and high operational tempos, multiple peer mentors will be selected within each squadron or workplace, thereby ensuring program accessibility and sustainment despite the transient nature of military life (e.g., temporary duty, permanent change of station, deployment). The total number of peer mentors selected for each unit is influenced by squadron size and mission demands. For example, units with 24-h operations and multiple work shifts would require peer mentors to be available during each work shift. Likewise, squadrons with multiple work locations would benefit from having peer mentors available in each work area.

Selected peer mentors complete a three-day training program focused on peer mentor certification, basic motivational interviewing skills, program curriculum, and crisis response planning. The training includes demonstration videos, role-play, and skills practice in order to facilitate skill development and mastery. Motivational interviewing principles and skills including (but not limited to) the use of open-ended questions, summary statements, decisional balance exercises, and readiness rulers [13] are included in the peer mentor training program because a central aim of Airman’s Edge is to enact behavior change, and motivational interviewing has extensive empirical support for its effect on increasing the likelihood of changing a wide range of health-related and risky behaviors [14,15].

Peer mentors are also trained to deliver educational content designed to target risk and protective factors associated with suicidal behaviors: sleep disturbance, social support and meaning in life, and safe firearm storage. This content is implemented at the unit level via existing communication channels including regularly scheduled commander’s calls, unit formations, workplace huddles, and wall hangings. The use of existing communication channels to deliver program content to the community instead of relying upon add-on delivery methods (e.g., training workshops, training seminars) minimizes interference with mission and operational demands and conforms to occupational safety approaches wherein risk mitigation concepts and strategies are integrated into the organization’s daily workflow. After their initial training, peer mentors meet on a regular basis with each other and with certified peer instructors to receive support, monitor and ensure program fidelity, troubleshoot barriers, and continually assess outcomes.

### 3.2. Program Design Elements

As the primary outcome of interest is suicidal behavior, Airman’s Edge was designed to incorporate P2P program features that are most strongly correlated with positive behavior change. The results of a recent review of P2P programs [10] suggest positive health-related behavior change is most probable when these programs include curriculum-based education and information provided by peer mentors as a group intervention (i.e., the peer educator model) combined with the provision of encouragement and informal types of support by peer mentors as a dyadic or one-to-one intervention (i.e., the peer support model).

### 3.3. Program Curriculum

Educational curriculum within Airman’s Edge cuts across multiple levels of the control hierarchy and focuses on three primary domains: (1) unit cohesion, purpose, and morale, (2) sleep quality, and (3) firearm storage.

#### 3.3.1. Unit Cohesion, Purpose, and Morale

Unit cohesion and meaning in life are positively correlated with overall psychological well-being and positive emotional states [16,17,18], but are negatively correlated with suicidal ideation [19,20]. Similar findings have also been reported in military samples [21,22,23,24,25]. Critically, this protective effect can be derived from the unit to which a service member is assigned. For example, service members are significantly less likely to report suicide ideation when they are assigned to units with people who report a high level of perceived social support [26]. Trauma-exposed service members also report less severe emotional distress when assigned to units with people who report experiencing positive emotions more frequently [27]. In both of those studies, unit-level effects were larger in magnitude than individual-level effects, suggesting that fostering a collective sense of cohesion and support, and building morale within a unit, may have a more powerful suicide prevention effect than changing how an individual service member is feeling. In Airman’s Edge, peer mentors are therefore trained to provide educational briefings that promote a sense of belongingness and collective purpose within their unit. Peer mentors also use materials developed by the Air Force’s Profession of Arms Center of Excellence that are designed to foster respect, appreciation, pride, and purpose (www.airman.af.mil/heritagetoday (accessed on 15 January 2020)). Improving morale and building a collective sense of support and a cohesive unit acts as an administrative control, changing the way service members interact within the workplace, and protecting them when exposed to hazards like occupational strain and trauma exposure.

#### 3.3.2. Sleep Quality

Sleep disturbance is a well-established risk factor for suicidal thoughts and behaviors [28]. Sleep disturbance may increase the risk of suicidal behaviors because it inhibits the ability to self-regulate emotions, to solve problems and process information, and to avoid or mitigate stress exposure [29,30] while increasing emotional reactivity and the likelihood of using use maladaptive behaviors in response to stressful situations [31]. Among military personnel and veterans, reductions in sleep disturbance have also been shown to precede reductions in suicide ideation and suicide attempts [32,33]. Sleep disturbance therefore serves as a critical hazard for suicidal behaviors. Of the many potential methods for reducing sleep disturbance, cognitive behavioral therapy for insomnia (CBT-I) has garnered the greatest degree of empirical support [34], and maintains its efficacy even when delivered in very low-intensity, community education formats [35]. In Airman’s Edge, peer mentors are trained to provide educational briefings and distribute information about CBT-I principles via handouts and fliers posted in the workplace. Educational briefings review common warning signs for fatigue, stages of sleep, appropriate napping strategies, and how to optimize ones environment to improve quality of sleep (i.e., control lighting, increase comfort, and reduce use of nicotine or caffeine). Teaching service members how to change their sleep-related behaviors to reduce the hazards associated with sleep disturbance corresponds to an engineering control, as this strategy is intended to remove sleep disturbance at its source, thereby reducing or eliminating service members’ exposure to this hazard.

#### 3.3.3. Firearm Storage

Firearms are the most common method of suicide among military personnel, accounting for nearly 70% of military suicides [2]. Multiple lines of evidence suggest that firearm availability is positively correlated with suicide mortality and limiting or restricting access to firearms is associated with lower suicide rates [36]. In the Israeli Defense Force, for instance, firearm-related suicides were reduced by 70% and overall suicides were reduced by 40% when a new policy restricting weekend access to government-issued firearms was implemented [37]. Storing firearms in safes and/or with a locking device is also correlated with lower suicide rates [38], implicating the potential value of strategies such as restricting weapons-bearing status, storing firearms in safes, using locking devices. Some data further suggest the protective effects of safe firearm storage may be more pronounced for individuals without mental illness or elevated suicide risk [39]. The benefits of safe firearm storage are therefore likely maximized if implemented as a routine practice across all military personnel who own firearms, not just those who are actively suicidal. In Airman’s Edge, Peer mentors will therefore be trained to provide informational briefings during regularly scheduled unit formations and workplace gatherings that encourage the adoption and use of firearm storage practices, and will make locking devices available to members of their unit who own or have access to firearms. These activities correspond to engineering controls because they serve to place barriers between service members and the hazard. Peer mentors will also be trained to promote social norms that support temporarily restricting a service member’s access to firearms when they are known to be severely distressed and/or suicidal, a strategy that corresponds to elimination controls because it involves physically removing the hazard.

#### 3.3.4. Crisis Response Planning

Peer mentors also receive training in crisis response planning and lethal means counseling to ensure they are prepared for face-to-face meetings with individual service members who may be experiencing heightened emotional distress and/or an acute suicidal episode. The crisis response plan (CRP) is a brief suicide prevention strategy that reduces the incidence of suicide attempts among military personnel by 76% and leads to faster reductions in suicide ideation and emotional distress as compared to typical suicide risk management strategies [5,40]. Typically handwritten on an index card, the CRP is a collaboratively developed plan that includes several key sections: personal warning signs that serve as indicators of emerging emotional distress, self-management strategies that work to reduce or distract from acute emotional distress, reasons for living or sources of meaning or purpose in life, sources of social support (e.g., peers, family members), and professional healthcare and/or crisis services. Initially developed and tested for use in mental health settings, the CRP can also be used by non-healthcare professionals as a concrete strategy for responding to acutely distressed individuals. CRP training is provided to peer mentors to prepare them to respond to fellow service members in crisis, akin to training non-healthcare professionals to use cardiopulmonary resuscitation and automated external defibrillators. To this extent, peer mentors will help promote local base resources to help normalize help-seeking behaviors of service members, and are trained to facilitate warm hand-offs with mental health professionals when warranted. The CRP is designed to protect a service member against the deleterious effects of life stressors, but does not necessarily eliminate, substitute, or isolate the service member from these stressors; it therefore corresponds to a PPE control.

## 4. Evaluating Program Effectiveness

A program evaluation of the Airman’s Edge P2P program is currently underway at Whiteman Air Force Base, Missouri. In this effort, a minimum of 1600 military and civilian personnel will be enrolled in the study. As the program is intended to effect change across the entire community, all personnel employed at the installation are eligible for enrollment, regardless of military or civilian status, branch of service, or military component. Specifically, this includes active duty military officers, enlisted personnel, and civilian employees of the department of defense that represent a diverse workforce of pilots, mechanics, security forces, medical personnel, administrators, and maintenance crews. The inclusion and exclusion criteria are purposefully broad to maximize generalizability to the military community. Personnel are eligible to participate if they are employed by the Department of Defense at the host installation, 18 years of age or older, and able to understand and speak the English language. The only exclusion criteria are an inability to understand and speak the English language and an inability to complete the informed consent process.

### 4.1. Planned Assessments

Participants complete a brief (i.e., 5–10 min) self-report web-based survey at baseline and 4, 8, 12, 16, and 20 months post baseline. To maximize participation and access, the survey can be completed using any computer or phone with internet access. This will help minimize assessment burden, which is an ongoing concern for military leaders. Primary outcomes include suicidal behaviors (which include suicide death, suicide attempts, aborted suicide attempts, and interrupted suicide attempts) and suicidal ideation. Suicide death will be assessed using DODSER data, and are treated as a subtype of suicidal behavior (i.e., suicidal behavior with fatal outcome). Suicide attempts, aborted suicide attempts, and interrupted suicide attempts are assessed using a combination of DODSER data and self-report data using items from the Self-Injurious Thoughts and Behaviors Interview-Revised (SITBI-R) [41]. Suicide ideation is also assessed using items from the SITBI-R. The SITBI-R assesses the occurrence of suicidal behaviors and ideation at any point during the target assessment period. As suicidal behaviors and suicidal ideation are assessed every four months, participants are asked to report if they have experienced any of these thoughts and behaviors during the 4 months preceding each assessment. Additional variables being assessed include cognitive flexibility, meaning in life, social support, depression, PTSD, alcohol and substance use, sleep disturbance, and more (see Table 1), collected via an online self-report survey. In addition to these self-report measures, installation-level administrative data (e.g., suicide deaths, reported suicide attempts) will also be analyzed.

### 4.2. Minimizing the Underreporting of Suicidal Thoughts and Behaviors

A key consideration for our study involved underreporting of suicidal thoughts and behaviors. Previous research has found that survey respondents, especially military personnel, are more likely to underreport suicidal thoughts and behaviors when their responses are identifiable [42,43,44]. Allowing for anonymous responding can increase self-disclosure of these constructs, but impedes our ability to track program effects on individual participants over time. To reconcile these competing demands, we therefore decided to use self-generated identification codes to link responses within individual participants across multiple assessments. Self-generated identification codes are created from the answers to a number of personally salient questions that yield low error rates and maximize the probability of successful matching over time (see Table 2) [45]. As compared to other anonymous data collection methods, self-generated identification codes provide higher quality data, reduce missingness, and increase the potential for matching responses, as high as 92.7% match rates, across time points [45].

### 4.3. Program Implementation Design

The study will roll out peer mentor training using a dynamic wait list design [46] with randomization occurring at the squadron level. The dynamic wait list design differs from traditional wait list designs primarily with respect to the timing of the intervention. In a traditional wait list design, half of the squadrons (i.e., N/2) would be randomized to implement the P2P program at the outset of the study and the remaining half would implement the P2P program later in the study. By contrast, a dynamic wait list design randomizes the timing of the intervention over the entire course of the study period. As Whiteman AFB has a total of 53 squadrons and units, 10–11 units are randomly selected to “switch” from the wait list to the P2P program during each time block, until all squadrons have implemented the P2P program.

The overall study design therefore constitutes a two-arm randomized clinical trial with several hypotheses (see Table 3). The program’s effects on suicidal thoughts and behaviors will be tested using multilevel, repeated measures generalized mixed effects regression models. Multilevel modeling is indicated due to the study’s nested design (repeated measurements nested within participants, participants nested within units) and our intention to consider outcomes at the participant and unit level. Mixed effects modeling will be used in order to model both fixed and random effects, as well as its ability to handle missing values, which are common and expected in longitudinal study designs. This is especially relevant to the proposed project because some participants are expected to miss assessment periods due to permanent change in station, temporary duty, deployment, and/or reassignment. Generalized models will be used because they are suitable for outcomes with a range of distributional properties including binary, Poisson, and zero inflated models, which are anticipated in the proposed study due to the nature of our primary outcomes (suicidal thoughts and behaviors), which are count variables.

Secondary analyses will include calculation of acceleration/deceleration of suicide rates at the installation level, and will examine associations among installation-level variables and suicide rates. This approach allows for the analysis of nonlinear temporal trends and is based on methods widely used in the science of applied behavior analysis for measuring and changing behavior.

## 5. Discussion

Suicide rates within military populations continue to rise, necessitating novel approaches that move interventions outside outpatient mental health clinics and into the general population. P2P programs are one way to facilitate a public health approach to reduce suicide rates within a specified organization. A recent report titled “A Report of Findings to Direct the Development of National Guidelines for Workplace Suicide Prevention,” recommends several factors that are in line with the proposed Airman’s Edge P2P program to include (1) improving community well-being by cultivating caring leaders, (2) increasing suicide awareness and how to help those in crisis, (3) providing coping strategies to improve self-care, (4) implementing means safety counseling, (5) identifying peer mentors, and (6) disseminating mental health and crisis resources [47]. With over two thirds of the American population actively engaged in a workplace environment, it is imperative that suicide intervention programs move out of traditional hospital and clinic confines and into workspaces.

The proposed Airman’s Edge P2P program is based on established work safety models that prioritize the physical elimination or removal of hazards from the environment [12]. This P2P program acts in a similar manner by shaping and redesigning the culture of the military community towards improved health and well-being, working to prevent suicide related incidences upstream of the crisis. This reduces the need for specialized intervention at the level of the individual, where resources can be scarce or stigma prevents individuals from seeking needed care. By intentionally building purpose, making meaning, cultivating unit cohesion, and improving morale this proposed P2P program for suicide prevention is aimed at eliminating working conditions that get in the way of employees enjoying a high quality of life within the workplace. A recent study of student nurses demonstrated that higher perceptions of social isolation were significantly correlated with both objective and subjective increase in an individual’s stress response [48]. Simple changes focused on shifting workplace culture potentially can have a significant impact on an individual’s wellbeing and quality of life. Additionally, teaching good sleep hygiene, emotion regulation skills, and safe firearm storage practices positions this P2P program in promoting health-enhancing skills and programs to encourage positive health-related behavior change.

Regarding strengths and limitations, this study uses a novel dynamic wait-list design that has several advantages within a transient military culture. As opposed to traditional wait-list designs, the dynamic wait-list designs mimics typical program rollout procedures in large institutions whereby program implementation is completed in smaller chunks compared to implementing the program across all divisions simultaneously. This design allows for improved generalizability of the results as it closer approximates real-world implementation procedures, should the program be successful and eventually adopted by the department of defense. Additionally, measuring Airman’s Edge with active duty service members on an active military installation is another strength of the study improving overall generalizability and ensuring culturally appropriate curriculum development.

Limitations consist of relying upon self-report data from Airmen, which at times is vulnerable to biased responding as a result of the sensitivity of the subject matter. To combat potential bias, the current study implemented self-generated identification codes to ensure anonymity of responses. In addition to individual self-report date, this study is collecting installation level data to examine change between installation level variables and base wide suicide rates. Analyses borrowed from the field of behavioral analysis used for measuring changing behaviors within an individual will be used to measure temporal change patterns within the installation. To further combat the biases of self-report, future research on P2P programs for suicide prevention should consider measuring objective measures, such as heart rate variability and other physiological measures.

## 6. Conclusions

To our knowledge, the Airman’s Edge project represents the first effort to rigorously evaluate a P2P program in any population for the purposes of suicide prevention. Results of this project will provide important clues for understanding the effects of P2P programs on suicidal thoughts and behaviors among military personnel in a real-world setting.

## Figures and Tables

**Table 1:** Individual variables and constructs measured.

Measure	Construct(s) Measured
**Outcomes: Suicide Thoughts and Behaviors**	
Suicide Death	Department of Defense Suicide Event Report
2. Self-Injurious Thoughts and Behaviors	Suicide ideation and planning, nonsuicidal self-injury, and suicide attempts (past four months)
**Mediators and Moderators**	
3. Cognitive Flexibility Inventory (Abbreviated)	Cognitive flexibility and emotion regulation
4. Interpersonal Needs Questionnaire (Abbreviated)	Social support
5. Meaning in Life Questionnaire (Abbreviated)	Purpose and meaning
6. Peer Mentor Program Exposure	P2P program utilization
**Covariates and Risk Factors**	
7. Patient Health Questionnaire, 2-item	Depression
8. PTSD Checklist (Abbreviated)	Posttraumatic stress
9. Alcohol Use Disorders Identification Test (Consumption Items)	Alcohol Use
10. Medication and Drug Use Questionnaire	Drug and medication misuse
11. Insomnia Severity Index	Sleep disturbance
12. Behavioral Risk Surveillance System Survey Questionnaire (Firearm Items)	Access to firearms
13. Mental Health Utilization	Use of mental healthcare services
14. Perceptions of Work	Emotional labor in the workplace; Perceptions of discipline and accountability
15. Home Safety Risk Assessment	Regular seat belt use and measures of driving while under the influence of alcohol

**Table 2:** Example of self-generated identification code.

	Question Stem: What Is the…					
	Month You Were Born?	Sex You Were Assigned at Birth, on Your Original Birth Certificate?	First Initial of Your First Middle Name?	First Letter of Your Mother’s or Female Caregiver’s First Name?	Number of Older Siblings (Brothers and Sisters) That You Have?	Self-Generated Identification Code
Example response Code Created	November 11	Female F	Jean J	Marjorie M	0 00	11FJM00

**Table 3:** Primary and secondary aims and hypotheses for Airman’s Edge P2P program.

Aim 1: To Test the Efficacy of a P2P Program for the Reduction of Suicidal Behavior among Military Personnel.
Hypothesis 1a: Military personnel randomized to the P2P condition will be significantly less likely to make a suicide attempt and will report significant reductions in suicide ideation during follow-up as compared to military personnel randomized to the control condition.
Hypothesis 1b: Squadrons randomized to the P2P condition will have significantly lower rates of suicidal behavior and suicide ideation as compared to squadrons randomized to the control condition.
Hypothesis 1c: Installation-level suicide rates will decelerate over time as the P2P program is implemented.
**Aim 2: To identify moderators and mediators of the P2P program’s effects on suicidal behavior.**
Hypothesis 2a: Military personnel randomized to the P2P condition will report larger improvements in emotion dysregulation and cognitive rigidity, meaning in life, and social support as compared to personnel randomized to the control condition.
Hypothesis 2b: Squadrons randomized to the P2P condition will report larger improvements in emotion dysregulation and cognitive rigidity, meaning in life, and social support as compared to squadrons randomized to the control condition.

## Data Availability

Not applicable.
